# Comparison between *Metschnikowia pulcherrima* and *Torulaspora delbrueckii* used in sequential wine fermentations with *Saccharomyces cerevisiae*

**DOI:** 10.3389/fmicb.2025.1590561

**Published:** 2025-07-02

**Authors:** Lisa Granchi, Francesca Patrignani, Angela Bianco, Giacomo Braschi, Marilena Budroni, Laura Canonico, Angela Capece, Anna Cauzzi, Maurizio Ciani, Fabio Chinnici, Valentina Civa, Luca Cocolin, Paola Domizio, Vasileios Englezos, Nicola Francesca, Carmela Gerardi, Francesco Grieco, Rosalba Lanciotti, Silvia Mangani, Carlo Montanini, Vincenzo Naselli, Giorgia Perpetuini, Rocchina Pietrafesa, Angela Racioppo, Gabriella Siesto, Rosanna Tofalo, Antonio Bevilacqua, Patrizia Romano

**Affiliations:** ^1^Department of Agriculture, Food, Environment and Forestry (DAGRI), Florence, Italy; ^2^Department of Agricultural and Food Sciences, University of Bologna, Bologna, Italy; ^3^Department of Agricultural Sciences, University of Sassari, Sassari, Italy; ^4^Department of Life and Environmental Sciences, Polytechnic University of Marche, Ancona, Italy; ^5^School of Agriculture, Forest, Food and Environmental Sciences (SAFE), University of Basilicata, Potenza, Italy; ^6^AEB SPA, Brescia, Italy; ^7^Department of Agriculture, Food, Environment and Forestry (DAGRI), University of Florence, Firenze, Italy; ^8^Department of Agricultural, Forest and Food Sciences, University of Turin, Grugliasco, Italy; ^9^Department of Agricultural, Food and Forest Sciences (SAAF), University of Palermo, Palermo, Italy; ^10^National Research Council, Institute of Sciences of Food Production (ISPA), Lecce, Italy; ^11^FoodMicroTeam s.r.l., Academic Spin-Off of the University of Florence, Florence, Italy; ^12^Department of Bioscience and Technology for Food, Agriculture and Environment, University of Teramo, Teramo, Italy; ^13^Department of Agriculture, Food, Natural Resources and Engineering (DAFNE), University of Foggia, Foggia, Italy; ^14^StarFInn s.r.l.s., Academic Spin-Off of the University of Basilicata, Potenza, Italy; ^15^Faculty of Economy, Universitas Mercatorum, Rome, Italy

**Keywords:** non-*Saccharomyces* yeasts, yeast growth, amino acids uptake, volatile organic compounds, phenolic profile, correlations

## Abstract

**Introduction:**

The interest toward the use of non-*Saccharomyces* yeasts in the winemaking process has been increasing because it has been demonstrated that they can contribute positively to the quality of wines; however, there is a gap in the literature on holistic approaches showing the effective contribution of non-*Saccharomyces* yeasts in sequential fermentations.

**Methods and results:**

Two commercial strains of *Metschnikowia pulcherrima* (Mp) and *Torulaspora delbrueckii* (Td) were used in sequential fermentations with *Saccharomyces cerevisiae* (Sc). The fermentations were monitored by evaluating cell viable counts, ethanol, glycerol, acids, amino acids, phenols, total antioxidant activity, total polysaccharides, and volatile organic compounds (VOCs). The results for amino acids pointed out after 2 days a lower utilization of amino acids by Sc per million of cells than Mp and Td; moreover, yeasts had a different preference hit. There were no significant differences in the final ethanol and glycerol content; however, the sequential fermentation Mp/Sc led to a significant decrease in malic acid levels, while the Td/Sc sequential fermentation resulted in a significantly lower acetic acid levels (13 mg/L vs 95–102 mg/L) and a higher phenol reduction. Finally, VOCs analysis showed differences in some compounds both after 2 days or at the end of fermentation (esters, and ketones, among others). Finally, both sequential fermentations resulted in a higher amount of polysaccharides.

**Conclusion:**

The findings of this research provide a basis for ensuring better management of sequential wine fermentation, and a possible approach for trials and data management.

## 1 Introduction

In recent years the interest toward the use of non-*Saccharomyces* yeasts in the winemaking process has been increasing because it has been demonstrated that they can contribute positively to the quality of wines, conferring different sensory characteristics to the final product (Aplin et al., 2021; Binati et al., 2020; Canonico et al., 2023; Comitini et al., 2021; Gobert et al., 2017; Tofalo et al., 2016; Tufariello et al., 2021; Wang et al., 2023). Indeed, non-*Saccharomyces* wine yeasts have some specific enological characteristics that are absent in *S. cerevisiae* species, and these can have additive effects on wine flavor and aroma (Romano et al., 2022a; Tufariello et al., 2021). For instance, mixed starter cultures with some specific non-*Saccharomyces* yeasts are reported to enhance the glycerol and polysaccharides content, to improve the aroma complexity, to reduce acetic acid and ethanol concentration (Contreras et al., 2014; Comitini et al., 2011; Domizio et al., 2014; Varela et al., 2016).

Among non-*Saccharomyces* yeasts, *Metschnikowia pulcherrima* is used in winemaking, especially thanks to its ability to grow in combination with *S. cerevisiae*, during the first stages of wine fermentation (Agarbati et al., 2023). Its positive effects include the synthesis of fruity and floral aromas (Canonico et al., 2019; Comitini et al., 2011; Rodriguez et al., 2010), and the biocontrol action (Vicente et al., 2020). Another non-*Saccharomyces* yeast which is attracting attention from the wine industry is *Torulaspora delbrueckii* because of its ability to enhance the complexity of wine aroma profile. It generally shows a higher β-glucosidase activity (Benito, 2018; Domizio et al., 2014; Zhang et al., 2021b), a reduced acetaldehyde content (Benito, 2018; Comitini et al., 2011; Fernandes et al., 2021; Ramírez and Velázquez, 2018; Renault et al., 2015), and enhanced levels of glycerol, mannoproteins, and positive aroma compounds (Comitini et al., 2011; Ramírez and Velázquez, 2018; Zhang et al., 2021b) and promotes malolactic fermentation (Agarbati et al., 2020; Benito, 2018).

The achievement of these positive effects on the overall quality of the wine is strictly dependent upon several factors, like the interactions between *S. cerevisiae* and non-*Saccharomyces* strains and the type of inoculum, i.e., simultaneous or sequential inoculum of the non-*Saccharomyces* strain, since during the alcoholic fermentation yeasts do not passively coexist but can establish positive or negative interactions. Different mechanisms can occur between yeast strains, including the production of inhibitory or toxic compounds, the modification of metabolism by quorum sensing or cell-to-cell contact (González et al., 2018; Kemsawasd et al., 2015; Mejias-Ortiz et al., 2023) and the nutrient competition (Evers et al., 2023; Rollero et al., 2018). Nutrient competition is one of the most studied mechanism, as it is responsible of reductions in the fermentative performance for amino acid removal (Su et al., 2020), inhibition for the production of toxic metabolites (Taillandier et al., 2014), or differences in the transcriptomic response (Mencher et al., 2021; Tronchoni et al., 2017).

Although there are many papers dealing with the sequential fermentation of non-*Saccharomyces* yeasts and *S. cerevisiae*, to the best of authors’ knowledge there is a literature gap that should be addressed in the field of sequential fermentation non-*Saccharomyces*/*S. cerevisiae*. First, the results are mainly based on enological performances, while there are a few data on other parameters, like phenols, amino acids, and polysaccharides. In addition, the experiments are generally based on a few batches (generally three), with a moderate dataset in term of amounts of data, while for a validation protocol it is important to generate a robust dataset, based on a significant number of independent samples and technical replicates. These two gaps were addressed in this research focusing on the enological performances of the two commercial non-*Saccharomyces* strains, *M. pulcherrima* (LEVULIA^®^PULCHERRIMA) and *T. delbrueckii* (LEVULIA^®^ TORULA), used in sequential fermentations with the commercial *S. cerevisiae* strain FERMOL^®^ Red Fruit in a commercial red grape juice in comparison with a fermentation conducted with the pure culture of *S. cerevisiae*. A robust protocol, not affected by the natural variability of data, was addressed by performing the fermentation trials in six different Research Units, belonging to the Italian Group of Microbiology of Vine and Wine (GMVV). In addition, a second research question was on possible correlations between cell count and some metabolisms, mainly amino acid consumption, to point out possible preference hit for different yeasts. Conducting experimental trials with these three commercial strains may have two important purposes. Firstly, it allows for the verification of the accuracy of the findings. Secondly, it provides valuable practical information for winemakers who want to use sequential fermentation in their winemaking process.

## 2 Materials and methods

### 2.1 Reagents and standards

Standards of kuromanin (cyanidin 3-O-glucoside chloride), oenin (malvidin-3-O-glucoside), rutin (quercetin 3-O-rutinoside), quercetin, catechin, caffeic acid, trans-resveratrol, trans-coutaric acid, caftaric acid, caffeic acid and kampherol-3-O-glucoside were from Extrasynthèse (Genay, France); Gallic acid, Folin–Ciocalteu phenol reagent, Trolox (6-hydroxy-2,5,7,8-tetramethylchroman-2-carboxylic acid), ABTS [2,2’-azino-bis (3-ethylbenzothiazoline-6-sulfonic acid)], mannan, HPLC grade acetonitrile and formic acid were supplied by Sigma-Aldrich (St. Louis, MO, United States). Ortho-phthaldialdehyde; mercaptoethanol; Na2B4O7⋅10H2O; dansylchloride; heptylamine; glutamic acid; asparagine monohydrate; glutamine; glycine; threonine; histidine; tyrosine; metionine; valine; phenylalanine; leucine; lysin monohydrochlorid and proline were purchased from Merck (Darmstadt, Germany). Aspartic acid and isoleucine were from Alfa Aesar Thermo Fisher Scientific (kandel, Germany). Cysteine hydrochloride monohydrate; arginine; serine and alanine were from MP Biochemicals (Eschwege, Germany). Tryptophan was obtained from Panreac Applichem (Darmstadt, Germany). 4-methyl-2-pentanol was from Merck (Milan, Italy).

### 2.2 Yeast strains

Three commercial strains, supplied by AEB (Brescia, Italy) as active dry yeast (ADY) and including one *S. cerevisiae* strain FERMOL^®^ Red Fruit (Sc) (Supplementary Table 1) and two non-*Saccharomyces* strains, *T. delbrueckii* LEVULIA^®^ TORULA (Td) (Supplementary Table 1) and *M. pulcherrima* LEVULIA^®^PULCHERRIMA (Mp) (Supplementary Table 1), were used in this study. In particular, for each yeast strain, the same batch was used in the experimental protocol in order to reduce the biological variability.

### 2.3 Grape juice

A commercial red grape juice (Quargentan, Verona, Italy) belonging to the same batch was taken into consideration and analyzed, showing the following main characteristics: sugars (glucose and fructose) (156.00 ± 3.60) g/L; malic acid, (3.72 ± 0.01) g/L; glycerol, (0.62 ± 0.04) g/L; total acidity, 6.90 g/L (expressed as tartaric acid); assimilable yeast nitrogen, (231.00 ± 9.33) mg/L (including amino acids, except proline, and ammonium), and pH 3.42 ± 0.02. The grape juice was added with 60 g/L of commercial grape sugar (Naturalia, Mazara del Vallo, Italy) to achieve 220 g/L of sugar. This grape juice was furnished to each Research Unit (RU) which assessed the absence of yeast or bacteria viable cells before performing the fermentation trials; no sulfur dioxide was added, as the juice was stabilized by the producer through pasteurization. No additive was added.

### 2.4 Protocol

**FIGURE 1:**
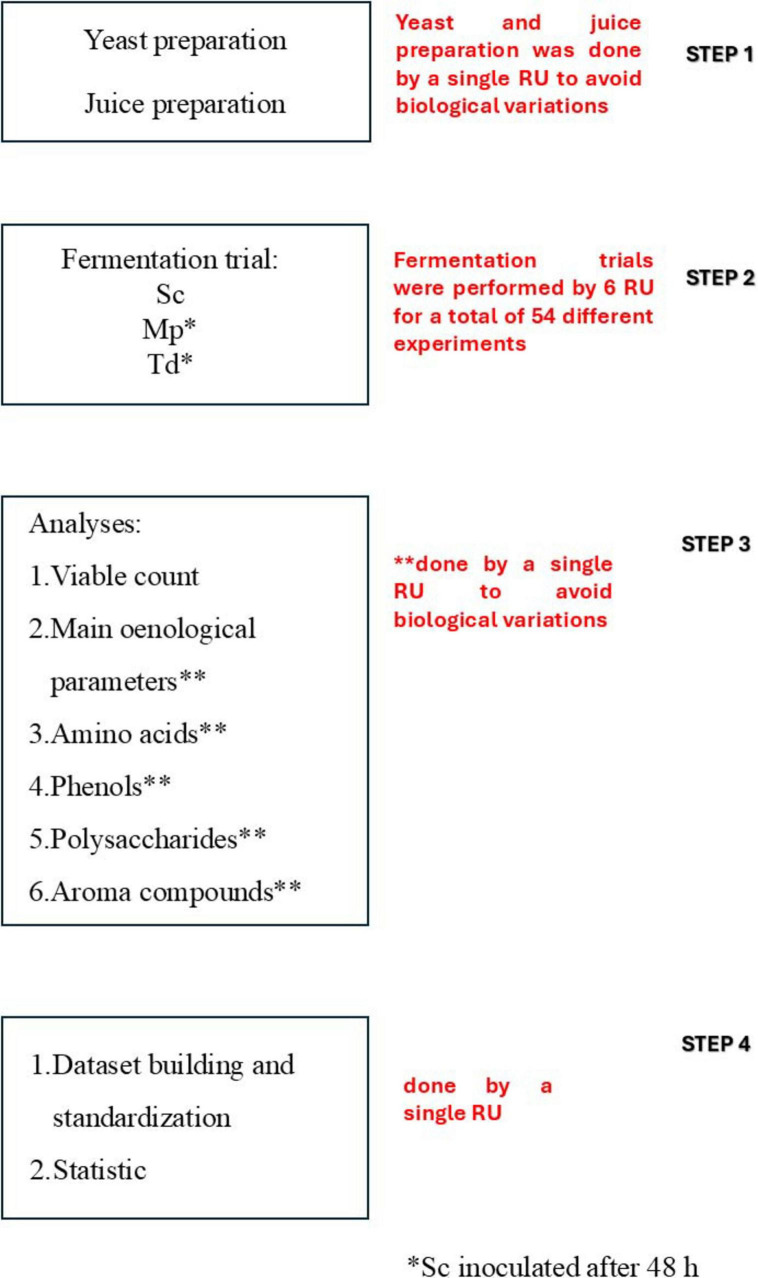
Overview of the protocol and planning of the research. RU, research unit; Sc, *Saccharomyces cerevisiae*; Mp, *Metschnikowia pulcherrima*; Td, *Torulaspora delbrueckii*.

The fermentation trials were performed simultaneously by six different RUs, belonging to the Italian Group of Microbiology of Vine and Wine (GMVV). The protocol (Figure 1) could be divided into four different steps:

Step 1: Juice and yeast cultures were prepared.

Step 2: Each RU performed the following fermentation trials: (1) Sequential fermentation with Mp and Sc strain inoculated after 48 h (three independent batches); (2) Sequential fermentation with Td and Sc strain inoculated after 48 h (three independent batches); (3) Pure fermentation with the Sc strain (three independent batches). In total 54 fermentation trials were accomplished.

Step 3: Each fermentation trial was monitored through microbial and chemical analyses; the samples were taken at different times during the alcoholic fermentation, whereas for chemical analyses, samples were taken at the beginning, after 48 h before the inoculum of Sc, and at the end of the alcoholic fermentation. Chemical analyses included the main enological parameters (residual sugars, ethanol, glycerol, acetic acid, malic acid), amino acids, volatile organic compounds (VOCs), phenols, total antioxidant activity and total polysaccharides.

STEP 1: Data analysis was performed by a single unit, focusing on dataset building, standardization and statistics.

### 2.5 Fermentation trials

The trials (step 2 of the protocol) were carried out in 500 mL flasks containing 350 mL of grape juice. Each yeast strain, belonging to the same batch, was rehydrated as described in the resolution OIV OENO 329/2009 (OIV, 2009), following the detailed procedure reported by Romano et al. (2022b). After rehydration, yeast culture was inoculated in the fermentation flasks to achieve a concentration of 2.00 × 10^6^ cells/mL in the juice. The inoculated flasks were sealed with Muller valves containing sulfuric acid, to allow only CO_2_ to escape from the system and weighed every day until the end of fermentation (as a constant weight for two consecutive days). All the fermentations were incubated at 20 ± 2°C in static conditions.

### 2.6 Microbiological analyses

Two different agar media were used: the differential substrate Wallerstein Laboratory Nutrient Agar (WL; Oxoid, Hampshire, United Kingdom) for total yeast count and the Lysine agar medium (LA; Oxoid, Hampshire, United Kingdom) for non-*Saccharomyces* yeast count. The latter medium containing lysine as the sole nitrogen source, prevents the growth of *S. cerevisiae* that does not usually use lysine. In detail, samples were taken at: T0: samples were collected just after the inoculum; T2: samples were collected 48 h after yeast inoculum. In sequential fermentations samples were collected before and after Sc inoculum; T4, T7, T10, and T15: samples were collected after 4, 7, 10, and 15 days of fermentation, respectively; Tf: samples were collected in correspondence of sugar exhaustion (< 2.0 g/L).

The plates were incubated at 26°C for 5 days and only those plates containing a statistically representative number of colonies were counted.

### 2.7 Main enological parameters

The general analytical parameters useful to characterize the fixed composition of grape juice and wines were evaluated using photometric readings in the mid-infrared regions, FTIR method, and the ultraviolet-visible region using the enzymatic approach. The samples were centrifuged at 5,000 × *g* at 4°C for 10 min and filtered at 0.20 μm through a Polyethersulfone membrane (VWR^®^, Darmstad, Germany). Titratable acidity, pH, and ethanol amount were measured according to the procedure described in OIV Resolution Oeno 390/10 All.2 (OIV, 2010) using a FOSS-WineScan™ Flex system (FOSS, Hillerød, Denmark). The principle of this technology is based on scanning the must or wine sample in the wavelength range of the mid-infrared wavelength range. Light is absorbed by the sample depending of the constituents present in the wine such as sugars or organic acids. This absorption value is translated into mathematical Fourier Transform model as a prediction of percentage concentration of the various constituents.

The values of L-malic, L-lactic, tartaric and acetic acids, together with reducing sugars, glucose and fructose, total sulfite and glycerol were measured using an iCubio iMagic M9 enzymatic analyser (Shenzhen iCubio Biomedical Technology Co., Ltd., Shenzhen, China). This system, equipped with autosampler and automated process management, allowed the sample and its reagent to be incubated at 37°C in a cuvette with 1 cm of optical pathway. The analytes were quantified by a spectrophotometric reading at 340 nm, except for tartaric acid which was detected at 520 nm after decolorization. The reagents used were: Enzytec™ Liquid Glycerol Cod. E8360, Enzytec™ Color Tartaric acid Cod. E3100; Enzytec™ Liquid D-Glucose/D-Fructose Cod. E8160; Enzytec™ Liquid D-glucose Cod. E8140; Enzytec™ Acetic acid Cod E1226; Enzytec™ Liquid D-Lactic acid Ref. No. E8245; Liquid L-Malic acid Cod E8280; Enzytec™ Liquid SO2-Total Cod E3100. The reagents and standards used were supplied by the manufacturer R-Biopharm AG (Darmstadt, Germany).

### 2.8 Amino acids

Amino acids in musts and wines were determined after derivatization with ortho-phthaldialdehyde-mercaptoethanol (OPA-ME) according to Kelly et al. (2010). The reaction mixture, prepared in 1.5 mL Eppendorf Safe Lock Tubes™, consisted of 100 μL of the sample (wine or grape juice), 20 μL of 20 mg/L heptylamine (internal standard, IS), 100 μL of OPA-ME solution (derivatization agent) and 0.2 M borate buffer at pH 9.5 up to a final volume of 400 μL. After 1 min, 800 μL of acetonitrile were added. The mixture was filtered at 0.45 μm and injected.

The derivatization reagent consisted of 500 μL of OPA solution (10 mg of OPA dissolved in 1 mL ethanol), 10 μL of mercaptoethanol and 1 mL of a 0.2 M borate buffer to pH 9.5 with sodium hydroxide). It was prepared daily and allowed to stabilize at room temperature for 90 min before use.

High Performance Liquid Chromatography (HPLC) determination of the AA OPA-derivatives was performed with an HPLC- UV/FLD, consisting of a ProStar 210 binary pump, a UV detector ProStar 340 (Varian Inc., Walnut Creek CA, United States) set at 262 nm, an 821-FP fluorescence detector (Jasco, Japan Spectroscopic co., Hachioji city, Japan) set at excitation and emission wavelengths of 345 and 455 nm, respectively, and a column oven Gecko 2,000 set at 40°C. Separation was carried out on a 150 mm × 4.6 mm × 5 μm Kinetex^®^ C18 column (Phenomenex Inc., Torrance CA, United States), protected by a C18 SecurityGuard^®^ cartridge with a flow rate of 1.0 ml/min. Mobile phase and gradient were those reported by Kelly et al. (2010).

Proline was quantified as dansyl-derivative as described by Tuberoso et al. (2015) using heptylamine as the internal standard. The reaction mixture consisted of 100 μL of sample (wine or grape juice), 10 μL of 100 mg/L heptylamine (IS), 100 μL of dansyl chloride solution (derivatization agent) and 0.2 M Na2B4O7⋅10H2O (pH 9.3) solution up to a final volume of 1,000 μL. The mixture was incubated for 30 min at 40°C in an ultrasonic bath and filtered at 0.45 μm before injection. Determination was carried out with an HPLC- UV/FLD Jasco series 4000 (Jasco, Japan Spectroscopic co., Hachioji city, Japan) equipped with a pump PU-4180, an autosampler AS-4050, a photodiode array detector MD-4010, a fluorescence detector FP-4025, and a column oven CO4060 equipped with a 150 mm × 4.6 mm x 5 μm Gemini^®^ C18 column (Phenomenex Inc., Torrance CA, United States) protected by a C18 SecurityGuard^®^ cartridge.

For both AA and proline, quantification was performed using calibration curves obtained according to the internal standard method which correlates the analyte/IS peak area ratio with concentration.

### 2.9 Total polyphenol content, antioxidant activity and HPLC phenolic profile

A rapid method (Magalhães et al., 2010) was used to assess the total phenol content of grape juices and wines using a microplate reader (Tecan, Infinite M200). Fifty microliters of Folin-Ciocalteu reagent diluted in water from Milli-Q system (1:5 v/v) were placed in each well of a microplate, and then 100 μL of sodium hydroxide solution (0.35 M) were added. The absorbance value at 760 nm was recorder after 5 min of incubation. Gallic acid was used to obtain a calibration curve in the range from 2.5 to 40.0 mg/L (R ≥ 0.9997). The total phenol content of each sample was expressed as Gallic Acid Equivalents (GAE).

Trolox Equivalent Antioxidant Capacity (TEAC) Assay was performed according to Gerardi et al. (2021). Briefly, 2,2’-azinobis (3-ethylbenzothiazoline-6-sulfonic acid) diammonium salt (ABTS, Sigma-Aldrich, St. Louis, MO, United States) radical cations were prepared by mixing an aqueous solution of 2.45 mM potassium persulfate (final concentration) and an aqueous solution of 7 mM ABTS (final concentration) and were allowed to stand in the dark at room temperature for 12–16 h, before use. The obtained ABTS radical solution was diluted in PBS (pH 7.4) to an absorbance value of 0.4 when read at 734 nm. A volume of 200 μL of ABTS radical solution was added to 10 μL of sample. Then, the absorbance at 734 nm was recorded after 6 min using the Infinite 200 Pro plate reader (Tecan, Männedorf, Switzerland). TEAC values were obtained considering the percentage inhibition with respect to Trolox used as a standard. TEAC values were expressed as Trolox equivalents (μmol/L). Magellan v7.2 software (Tecan, Männedorf, Switzerland) was used to control the plate reader.

A reversed phase-HPLC analytical method was used for the analysis of phenolic compounds. The apparatus was an Agilent-1100 liquid chromatograph (Agilent Technologies, Italy) equipped with a DAD detector (Agilent 1260 Infinity) and the separation was performed on a C18 column (5 μm UltraSphere 80 Å, 4.6 i.d. × 250 mm length) following the conditions described by Gerardi et al. (2021). Chromatograms were acquired at 520, 280, 320, 370, and 306 nm. Identification of compounds was based on the comparison of peak retention time with the retention time and UV vis spectra of pure standards while quantification was performed by adopting the external standard method.

### 2.10 Polysaccharides

Polysaccharides quantification was carried out according to Portaro et al. (2022). In detail, the samples were filtered (0.45 μm acetate cellulose membranes) and directly injected (20 μL) into the HPLC apparatus (Varian Inc., Palo Alto, CA, United States) equipped with a 410 series autosampler, a 210 series pump, and a 356-LC refractive index detector. Isocratic separation was performed on a TSK G-OLIGO-PW (808031) column (30 cm × 7.8 mm i.d.) and a TSK-GEL OLIGO (808034) guard column (4 cm × 6 mm i.d.) (Supelco, Bellefonte, PA, United States). The mobile phase was 0.2M NaCl, at a flow rate of 0.8 mL/min. Peaks were quantified by comparison with an external calibration curve of mannan from 50 to 1,000 mg/L. The analysis of the peaks was performed using the software Galaxie Chromatography Data System (version 1.9.302.530) (Varian Inc., Palo Alto, CA, United States).

### 2.11 Volatile organic compounds

Volatile organic compounds were qualitatively and quantitatively evaluated by head space solid phase microextraction using a gas chromatograph combined with a mass spectrometer detector Shimadzu QP2010 (Shimadzu, Milan, Italy). A Carboxen^®^/DVB/PDMS, 50/30 μm fiber (Supelco, Merck, Milan, Italy) was used to perform the solid phase microextraction (SPME). The samples (5 mL) were placed in vials with 1 g of NaCl and incubated for 10 min at 45°C. Then the fiber was exposed to the vial headspace for 30 min at 45°C. The volatile molecules adsorbed were transferred in the GC injector port in splitless mode at 250°C for 10 min. The column used was Zebron ZB-WAX (30 m × 250 μm × 1.2 μm) (Phenomenex, Milan, Italy). The oven initial temperature was 50°C for 1 min and then increased by 4.5°C/min up to 200°C for 10 min. Gas-carrier was helium at 1.0 mL/min flow.

For each head-space compound eluted from the chromatographic system, identification was performed by comparing the corresponding average mass spectrum with the NIST08 mass spectra library using the GCMS post-run analysis software version 4.44 (Shimadzu, Milan, Italy), employing an algorithm that includes Kovats index correction, while quantification was performed using the internal standard method with 4-methyl-2-pentanol as calibrator compound at a final concentration of 6 mg/L by comparison between each compound area and standard peak area. Quantification results were expressed as equivalent mg/L (mg/L eq.). For each detected compound, the mg/L eq. represents the amount of compound present in the headspace in dynamic equilibrium with the aqueous phase. Analyses were performed in triplicate.

### 2.12 Statistical analyses

The analyses were performed on 18 independent batches (six research units and three batches for each unit); data were preliminary analysed to assess the basic requisite of homoscedasticity and standardized (for sugar, amino acids, acids, and phenols) as reduction (decrease of amount compared to the unfermented juice). Significant differences were pointed out through one-way or multi-parametric Analysis of Variance (ANOVA), using Tukey’s test as the *post hoc* multiple comparison test (*P* < 0.05), when data followed a normal distribution, or *t*-test for paired comparisons, and the non-parametric test of Friedman when data did not show homoscedasticity. Clustering and sample grouping were done through two-way joining (heat map) or Principal Component Analysis, using the single Euclidean distance as the amalgamation method, while the significance of the correlation was evaluated through the Pearson coefficient. Statistic was done by the software Statistica for Windows, ver. 12.0 (Statsoft, Tulsa, OK, United States).

## 3 Results and discussion

### 3.1 Yeast population kinetics

The yeasts used in this research are strains commercially available; thus, their properties and characteristics, at least under controlled conditions, are well-known, and this is an important requisite to assess the correctness of findings in experimental works with a wide number of parameters, as done for this paper. In addition, the use of these strains is in line with a research question, that is to show how to design a validation protocol and to offer valuable practical information for winemakers interested in employing sequential fermentation in their winemaking process, especially considering the lack of published studies on these strains. Another choice relies upon the use of commercial grape juice, due to the fact that the main goal of the research was to focus on yeas behavior; therefore, the use of the same medium avoids a possible confounding effect due to the matrix and its variability. The lack of additives also avoids the risk of possible impacts on fermentation kinetics.

**FIGURE 2:**
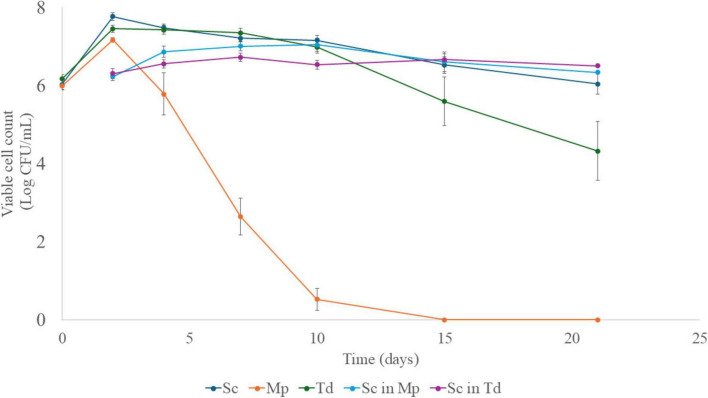
Viable counts of *S. cerevisiae*, *M. pulcherrima* and *T. delbrueckii* (mean values ± standard error). Sc in Mp, *S. cerevisiae* in sequential fermentation with *M. pulcherrima*; Sc in Td, *S. cerevisiae* in sequential fermentation with *T. delbrueckii*; Sc, *S. cerevisiae* pure culture; Mp, *M. pulcherrima*; Td, *T. delbrueckii*.

Figure 2 shows the growth kinetics of Sc in pure culture and during the sequential fermentations with Mp and Td. The population of the pure culture of Sc increased in the first 2 days of fermentation up to 7.80 log CFU/mL, then it decreased steadily, reaching 6.00 log CFU/mL at the end of fermentation. In the sequential fermentations, Sc inoculated after Mp and Td showed lower cell concentrations (7.00 and 6.50 Log CFU/mL, after 10 and 7 days, respectively). In contrast, in the sequential fermentation with Td, Sc did not show a significant reduction. Moreover, in the sequential fermentation with Mp, after reaching the maximum value, Sc exhibited a 1.0-Log reduction. Some other authors have found no difference in the growth of *S. cerevisiae* during sequential fermentation with *M. pulcherrima* compared to pure culture (Aplin et al., 2021; Binati et al., 2020; Canonico et al., 2023; Dutraive et al., 2019). Nevertheless, the growth inhibition of *S. cerevisiae* by *M. pulcherrima* was demonstrated by an *in vitro* assay to be due to a fast removal of iron (Melvydas et al., 2020). Moreover, it is noteworthy to highlight that interactions among yeast species might be strain-dependent (Roca-Mesa et al., 2022; Wang et al., 2016).

Taillandier et al. (2014) found lower cell concentrations of *S. cerevisiae* in sequential fermentations with *T. delbrueckii*, thus confirming the results of the present research. The authors supposed that this effect could be due to the consumption of assimilable nitrogen in must by *T. delbrueckii* during the first 48 h. However, Zhu et al. (2021) found that *S. cerevisiae* QA23 strain attained lower values of cell count with the non-*Saccharomyces* yeast, unless nitrogen had been added before *S. cerevisiae* inoculation. Therefore, it could be suggested the existence of other interaction mechanisms, such as possible competition for vitamins and negative cell-cell contact (Petitgonnet et al., 2019). Nevertheless, in this study in the presence of Td, the Sc strain maintained the initial cell concentration throughout the fermentation process, while, in pure culture, it decreased by 2-Log units. This longer survival could be caused by the death and autolysis of the non-*Saccharomyces* yeast (Binati et al., 2020).

Concerning the growth of the other two non-*Saccharomyces* yeast (Mp and Td) (Figure 2), Mp reached the maximum population in the first 2 days of fermentation before inoculating Sc. Then, the viable cells decreased up to 2.50 × 10^3^ CFU/mL after 7 days and were below the detection limit after 10 days. The sudden reduction of Mp after the inoculation of Sc is in agreement with the results obtained by several other authors (Binati et al., 2020; Comitini et al., 2011; Dutraive et al., 2019; Seguinot et al., 2020), and likely related to some yeast metabolites such as ethanol, killer toxins, and peptides or nutrient limitation and cell-to-cell contact (Dutraive et al., 2019). On the contrary, Td, after attaining at day 2 the maximum cell density of 7.20 log CFU/mL, exhibited an 8 days stationary phase and then decreased up to 4.30 log CFU/mL after 21 days.

*M. pulcherrima* and Td were not tested as single cultures in this research. Although it could be useful to focus on their growth and metabolic activity as pure cultures, the main goal of this paper was on the scalability and validation of effective protocols. During commercial processes, non-*Saccharomyces* yeasts are not used to perform pure fermentation as they do not achieve sugar consumption, thus they are not comparable with single *Saccharomyces* fermentations.

### 3.2 Main enological parameters of wine

After 2 days of fermentation, Sc consumed about 48.19% of glucose and 26.74% of fructose, confirming its glucophilic behavior (*P* < 0.01). In contrast, both non-*Saccharomyces* yeast strains showed no significant differences between glucose and fructose consumption and utilized only about 30% and 40% of the sugars (glucose + fructose) (data not shown). The lower sugar consumption of both non-*Saccharomyces* yeast species compared to *S. cerevisiae* seems to be in part a result of their respiratory metabolism of carbon sources during the early stages of winemaking (Brandam et al., 2013; Maicas and Mateo, 2023). *M. pulcherrima* is a Crabtree negative yeast (Schnierda et al., 2014) and possesses a complete electron transport chain, including complexes I, III, and IV as reported by Pettersen et al. (2023). Therefore, it consumes sugars by respiration rather than fermentation during the first fermentation stages. This metabolic process, which is more efficient in producing ATP than alcoholic fermentation, reduces sugar influx into the yeast cells.

In addition, *T. delbrueckii* is missing paralog genes encoding glyceraldehyde-3-phosphate dehydrogenase, enolase and pyruvate kinase and that in the fermentation pathway, *T. delbrueckii* expressed only one pyruvate decarboxylase gene (Tondini et al., 2019). Consequently, the concentrations of ethanol and glycerol were significantly lower in samples fermented by Mp and Td after 2 days (Table 1). Nevertheless, at the end of the fermentation trials, sugars were completely consumed and no differences in the final ethanol and glycerol content were observed. Literature reports controversial data for ethanol content in sequential fermentations; for example, González-Royo et al. (2015) used *T. delbrueckii* Biodiva™ and *M. pulcherrima* Flavia^®^ as commercial strains and did not find differences in ethanol content. On the other hand, other authors observed ethanol reduction for sequential fermentations with *M. pulcherrima* and *T. delbrueckii*, with concentrations ranging from 0.2% to 1% (v/v) (Hranilovic et al., 2020; Mun oz-Redondo et al., 2021). Concerning glycerol concentration, Mun oz-Redondo et al. (2021) found no statistical differences in sequential fermentation with *M. pulcherrima* while in sequential fermentations with *T. delbrueckii*, the wines presented about 1.3 g/L higher content of glycerol. Nevertheless, other authors have reported no differences in glycerol production by *T. delbrueckii* (Benito, 2018).

**TABLE 1:** Ethanol, glycerol, and acetic acid produced, and malic acid consumed throughout fermentation (mean values ± standard error) after 2 days (T2) and at the final time of fermentation (Tf).

	Days of fermentation					
	T2			Tf		
	Mp	Td	Sc	Mp	Td	Sc
Ethanol (%, vol/vol)	1.73 ± 0.12a	2.53 ± 0.15b	4.61 ± 0.38c	12.31 ± 0.05d	12.36 ± 0.02d	12.43 ± 0.01d
Glycerol (g/L)	0.57 ± 0.09a	0.99 ± 0.18a	2.88 ± 0.27b	6.11 ± 0.20c	5.74 ± 0.31c	5.26 ± 0.13c
Acetic acid (mg/L)	9.61 ± 5.26a	11.44 ± 4.46a	67.17 ± 14.40b	101.94 ± 17.23b	12.83 ± 6.15a	95.17 ± 21.92b
Malic acid consumption (g/L)	0.42 ± 0.04a	0.45 ± 0.04a	0.61 ± 0.05b	1.36 ± 0.03d	1.05 ± 0.02c	0.98 ± 0.03c

Sc, pure fermentation of *S. cerevisiae*; Td, sequential fermentation *T. delbrueckii-S. cerevisiae*; Mp, sequential fermentation *M. pulcherrima-S. cerevisiae*. Letters indicate significant differences in a row (one-way ANOVA and Tukey’s test, *p* < 0.05).

At the end of fermentation, the amount of acetic acid was 12.83 ± 6.15 mg/L for Td, 95.17 ± 21.92 mg/L for Sc, and 101.94 ± 17.23 mg/L for Mp (Table 1), thus confirming previous findings reporting lower levels of volatile acidity in mixed fermentation *T. delbrueckii*/*S. cerevisiae* (Agarbati et al., 2020; Azzolini et al., 2012; Comitini et al., 2011). Tondini et al. (2019) reported that the low acetic acid production by *T. delbrueckii* is consistent with alcohol dehydrogenase transcripts (responsible for converting acetaldehyde to ethanol) higher than aldehyde dehydrogenase transcripts (the enzyme involved in acetic acid production). Since acetic acid is responsible for the negative attribute of volatile acidity in wine, sequential fermentation with *T. delbrueckii* can be a useful tool to reduce this undesirable compound. Considering that some studies have found a malic acid reduction in sequential fermentations with non-*Saccharomyces* yeasts, the concentrations of this acid were assessed. After 2 days of fermentation, Td and Mp exhibited a significantly lower (*P* < 0.05) reduction of malic acid than Sc (0.42/0.45 vs. 0.61 g/L) (Table 1). At the end of fermentation, a further decrease of malic acid of 1.05–1.36g/L was observed. This is in agreement with the removal of malic acid by *M. pulcherrima* found by Du Plessis et al. (2017), Canonico et al. (2023) and by *T. delbrueckii* according to Belda et al. (2015), Mun oz-Redondo et al. (2021). Malic acid consumption during alcoholic fermentation may be particularly advantageous in red wine production, as malolactic fermentation is typically desired.

### 3.3 Amino acid consumption

**FIGURE 3:**
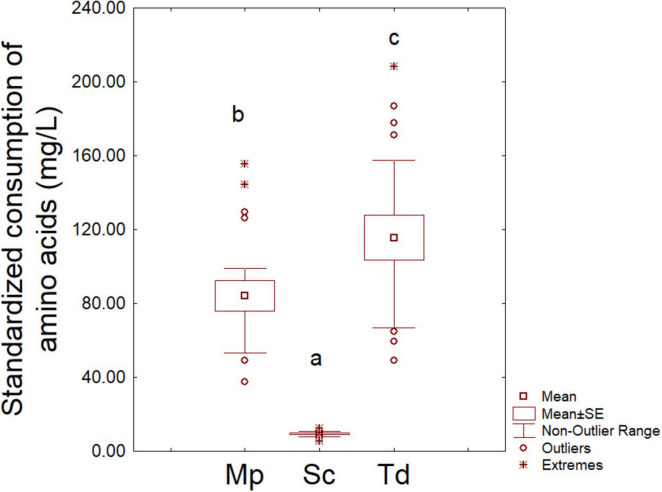
Amino acid consumption per million cells after 2 days. Sc, *S. cerevisiae*; Mp, *M. pulcherrima*; Td, T. *delbrueckii*. Letters indicate significant differences (one-way ANOVA and Tukey’s test, *P* < 0.05).

After 48 h of fermentation, amino acid consumption was significantly higher in Sc pure fermentation (724.17 ± 21.20 mg/L) than Mp and Td (143.11 ± 30.40 and 242.83 ± 32.30 mg/L, respectively), thus suggesting a higher consumption of amino acids by Sc (data not shown). This trend was the result of a higher cell concentration of Sc than Mp and Td, as aforementioned; therefore, as a second step, the amount of consumed amino acids was standardized per million of cells (6 log CFU/mL) to avoid the confounding effect of cell concentration (Figure 3). In this graph Sc showed a more homogeneous trend with a mean value of amino acid consumption/million cells of 9.33 mg/L, while the mean values for Mp and Td were respectively, 84.18 and 115.63 mg/L. To authors knowledge, this is the first time that this kind of approach (amino acid consumption standardized per amount of cells) has been proposed, thus there are no literature reports in this field.

**FIGURE 4:**
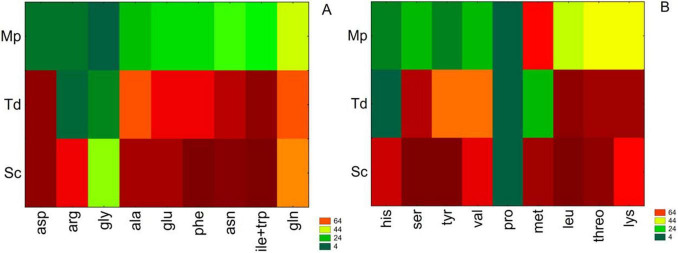
Two-way joining for amino acid consumption (%) after 2 days. Sc, *S. cerevisiae*; Mp, *M. pulcherrima*; Td, T. *delbrueckii*. **(A)** Asp, aspartate; Arg, arginine; Gly, glycine; Ala, alanine; Glu, glutamate; Phe, phenylalanine; Asn, asparagine; Ile + Trp, isoleucine + tryptophan; Gln, glutamine; **(B)** His, histidine; Ser, serine; tyr, tyrosine; val, valine; Pro, proline; Met, methionine; Leu, leucine; Threo, threonine; Lys, lysine.

After focusing on the total consumption of amino acids, each compound was separately analyzed, as reported in Supplementary Table 2. As shown by the table, significant differences were recorded after 2 days. Then, these results were further standardized as consumption percentages and treated through a two-way joining approach (Figure 4).

According to the preferred amino acids, four groups were individuated: group A corresponds to the most used amino acids (≥ 80%), B to intermediate assimilation (≥ 60%), C to low assimilation (≥ 40%) and D indicates very low levels of assimilation (≤ 20%). The amino acid uptake was yeast/strain dependent. After 48 h in Mp the highest uptake value was recorded for methionine (66%) followed by threonine, lysine, glutamine (about 45%) and leucine, asparagine, isoleucine+tryptophan (about 35%). The remaining amino acids were assimilated to a lesser extent. Td displayed a higher uptake (> 80%) of 6 amino acids (aspartate, leucine, isoleucine, tryptophan, lysine, and threonine) followed by other 8 amino acids consumed in a range between 58% and 79% (asparagine, serine, glutamate, alanine, phenylalanine, glutamine, tyrosine and valine) whereas 4 amino acids (arginine, methionine, glycine and histidine) were scarcely or not assimilated. On the contrary, Sc in pure culture assimilated most of the amino acids.

The results demonstrated that Mp and Td, by consuming specific amino acids during the first 2 days, strongly modified the quantity and characteristics of the nitrogen sources initially present in the grape juice, then available for the subsequent fermentation carried out by Sc. These findings are in agreement with those of other studies (Gobert et al., 2017; Prior et al., 2019; Seguinot et al., 2020; Taillandier et al., 2014). Nevertheless, it is important to underlying that the assimilation profile, as well as the concentration of the amino acids, may be strictly strain-dependent as well as affected by the fermentation conditions, such as temperature and initial nitrogen amount occurring in grape juice (González-Royo et al., 2015; Seguinot et al., 2020). Td consumed 14 amino acids at higher levels than Mp, strongly affecting the grape juice amino acids profile. Among the consumed amino acids by Td, threonine, isoleucine, leucine, tryptophan and methionine are involved in the Ehrlich pathway to produce higher alcohols. Hence, the growth of this non-*Saccharomyces* yeast in the first stages of fermentation can contribute to the wine aroma complexity.

### 3.4 Phenols and polysaccharides

The evaluation of phenolic profile and antioxidant potential is an important requisite for the overall quality of wine; mainly, phenols contribute to the ability to withstand oxidative changes over time, while antioxidant potential is an indirect evaluation of polyphenols to neutralize damaging free radicals, thus preserving color intensity and clarity (da Silva Duarte et al., 2024). In addition, phenolic profiles and antioxidant profiles are distinctive traits of the yeast strains (da Silva Duarte et al., 2024).

**FIGURE 5:**
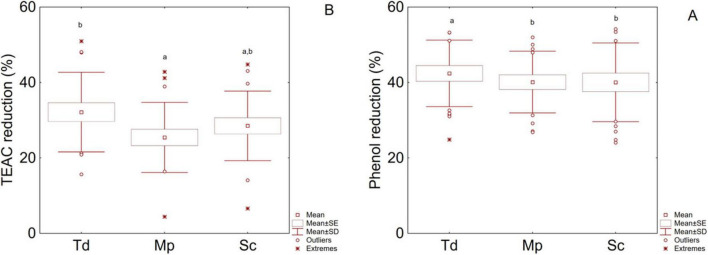
Box-whiskers plot for the effect of fermentation on phenol reduction **(A)** and antioxidant potential reduction (TEAC) **(B)**; letters indicate significant differences (one-way ANOVA and Tukey’s test, *P* < 0.05). Sc, pure fermentation of *S. cerevisiae*; Mp, sequential fermentation *M. pulcherrima/S. cerevisiae*; Td, sequential fermentation *T. delbrueckii/S. cerevisiae*.

Figure 5 summarizes the percentage decrease of total phenols (TP) (A) and antioxidant activity (TEAC) (B) during the fermentation. All fermentations caused a decrease of TP (39%–42%) and TEAC (26%–32%), with the highest decrease percentage measured in Td (42% for phenol and 32% for TEAC) and the lowest one for Mp (40% for phenol and 25% for TEAC). Sc also showed the lowest reduction value for phenols and an intermediate trend for TEAC.

Also individual polyphenols were identified and quantified in wines obtained through sequential fermentation and by Sc pure culture (Supplementary Table 3). Quercetin, rutin and catechin showed similar concentrations in grape juice as well as at the end of fermentation. Quercetin-3-O glucoside, Kempferol-3-O-glucoside, trans-caftaric acid, trans-coutaric acid and anthocyanins content showed a decrease at the end of fermentation in all fermented samples. Gallic acid and caffeic acid concentrations increased at the end of fermentation and were higher in sequential fermentation with Mp and in Sc. A higher gallic acid content can improve the color quality and stability of red wine and enhance the preservation of white wine due to its antioxidant activity.

*S. cerevisiae* starter strains are routinely employed in winemaking and a recent investigation suggested the role of autochthonous selected yeast strains to enhance the amount of the phenolic compounds and, consequently, the antioxidant and inflammatory activity in the produced wine (Grieco et al., 2019). However, Escribano-Viana et al. (2019), da Silva Duarte et al. (2024) have recently assessed that the polyphenolic profile of wine can be modulated with the use of a specific non-*Saccharomyces* fermentation starter. Concerning the polyphenolic profile of produced wines, Miranda et al. (2023) reported similar loss of total polyphenols and antioxidant activity percentage during fermentation of Tinta Negra red grape variety inoculated with five non-*Saccharomyces* yeasts strains.

It is reasonable to assume that the effect on the phenolic profile was the result of specific enzymatic activity or of different adsorption by the cell walls, in particular due to physicochemical interactions with mannoproteins (Caridi et al., 2004; Nguela et al., 2016, 2023; Zhang et al., 2021a). Hence, the increased gallic acid content might depend on the hydrolysis of tannins by tannase release by yeasts (Lopes et al., 2018), while the increase of caffeic acid might be related to hydrolysis of trans-caftaric acid, the ester of tartaric acid and caffeic acid (Zhang et al., 2021a). Yet, the different concentrations of the phenolic compounds, observed among the trials, carried out by the different yeast strains at the end of the fermentation, might be due to different interactions with the yeast cell wall mannoproteins and in turns with the different cell wall composition generally recognized at the genera, species, and strain level (Domizio et al., 2014).

Polysaccharides were also assessed; it is well-known that yeasts release polysaccharides, mainly mannoproteins, but their amount and characteristics depend on the growth conditions of the yeast and on the yeasts (Domizio et al., 2014). During the trials, higher amounts of polysaccharides were found for the sequential fermentations (about 343.00 ± 6.67 mg/L), while a lower value was found in Sc (316.00 ± 6.13 mg/L). These results are in agreement with those found by other authors (Comitini et al., 2011; Domizio et al., 2014) and, taking into account the impact of mannoproteins on the wine color stability (Escot et al., 2001) they could suggest a possible role on phenol profile.

### 3.5 Volatile organic compounds

In recent decades, non-*Saccharomyces* yeasts have undergone a positive reassessment in enology due to their capacity to synthesize aromatic compounds during the early stages of alcoholic fermentation, thereby contributing significantly to the complexity of wine (Sant’Ana and Lemos Junior, 2024; Romano et al., 2022a). These yeasts are now regarded as valuable biotechnological agents, particularly in co-fermentation with *S. cerevisiae*, owing to their distinct metabolic and enzymatic activities that influence the development of the wine’s aromatic profile. According to Sant’Ana and Lemos Junior (2024), non-*Saccharomyces* yeast species, mainly *T. delbrueckii* and *M. pulcherrima*, are increasingly being utilized for their potential to enhance wine complexity through the development of more nuanced sensory profiles (Englezos et al., 2022; Lemos Junior et al., 2016). Moreover, these yeasts can modulate critical enological parameters—including acidity, residual sugar, and ethanol concentration—thereby improving aromatic richness and textural quality without the need for synthetic additives (Benito, 2018). This approach aligns with evolving consumer preferences for more natural products and supports the broader objective within the wine industry to minimize the environmental footprint of production practices.

The effect of the sequential fermentations on the sensory was assessed through VOC quantification; however, due to the complexity of dataset a preliminary one-way ANOVA of VOCs was done to point out the compounds showing a significant contribution and only these VOCs were used to run a Principal Component Analysis (PCA).

**FIGURE 6:**
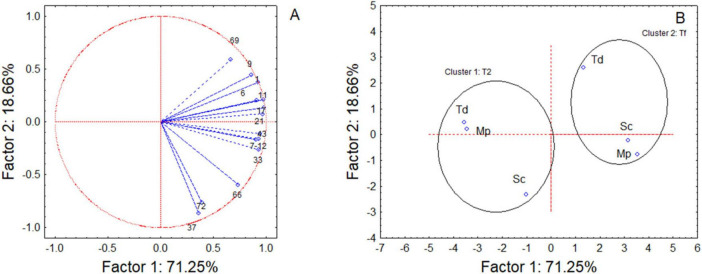
Variable **(A)** and Case **(B)** projection for Principal Component Analysis run on some representative volatile compounds. See Table 2 for the correspondence number vs compound. Sc, pure fermentation of *S. cerevisiae*; Td, sequential fermentation *S. cerevisiae-T. delbrueckii*; Mp, sequential fermentation *S. cerevisiae-M. pulcherrima*. T2, after 2 days; Tf, final time of fermentation.

The analysis allowed to explain 89.91% of the total observed variability (Figure 6); along Factor 1, samples grouped into two well-defined clusters depending on fermentation time, while on Factor 2, samples were differentiated based on the starter culture. After 2 days of fermentation, within cluster 1, Mp and Td clustered closely together, distinct from the samples Sc. In addition, a focus on the effects of variables, revealed a differential accumulation of VOCs (Figure 6 and Table 2). In fact, Sc exhibited the highest concentration of all the identified compounds in the headspace, including ethyl acetate (25.04 mg/L eq.), ethyl caproate (1.30 mg/L eq.), ethyl caprylate (4.36 mg/L eq.), ethyl caprate (2.80 mg/L eq.), β-phenylethyl acetate (2.24 mg/L eq.), acetic acid (3.14 mg/L eq.), caproic acid (5.36 mg/L eq.), caprylic acid (25.12 mg/L eq.), and β-phenylethanol (3.91 mg/L eq.).

**TABLE 2:** Selected volatile organic compounds (mg/L eq.) after 2 days (T2) and at the final time of fermentation (Tf).

Chemical class	Code	Compound	mg/L eq.											
			Days of fermentation											
			T2						Tf					
			Mp		Td		Sc		Mp		Td		Sc	
Ester	1	Ethyl acetate	20.45	B	15.16	A	25.04	C	76.85	A	85.72	B	79.74	A
	21	Ethyl caproate	0.01	A	0.20	B	1.30	C	15.94	C	8.55	A	13.23	B
	33	Ethyl caprylate	-^1^	A	0.14	A	4.36	B	15.59	C	4.25	A	12.09	B
	43	Ethyl caprate	0.01	A	0.31	A	2.80	B	7.61	B	4.06	A	4.49	A
	61	β-Phenylethyl acetate	0.26	A	0.65	B	2.24	C	14.71	C	10.61	B	7.89	A
Ketones	7	Methyl isobutyl ketone	1.15	A	1.01	A	1.41	B	3.69	B	1.71	A	3.95	B
	13	Amyl methyl ketone	1.03	A	2.09	B	11.37	C	75.03	B	62.70	A	78.81	B
Alcohols	9	1-propanol	0.52	A	1.85	B	4.28	C	11.46	A	17.36	C	13.47	B
	12	Isobutyl alcohol	4.29	B	1.80	A	8.87	C	15.94	B	9.98	A	21.60	C
	17	Isoamyl alcohol	10.91	A	15.64	B	35.19	C	75.04	B	70.59	A	86.65	C
	69	β-Phenylethanol	3.04	B	0.89	A	3.91	B	6.12	A	14.79	C	9.21	B
Volatile fatty acids	37	Acetic acid	2.16	B	0.89	A	3.14	C	2.98	B	0.71	A	2.67	B
	66	Caproic acid	0.24	A	1.04	B	5.36	C	4.97	B	2.38	A	4.56	B
	72	Caprylic acid	0.63	A	4.11	B	25.12	C	13.75	B	5.73	A	13.02	B

Sc, pure fermentation of *S. cerevisiae*; Td, sequential fermentation *T. delbrueckii-S. cerevisiae*-; Mp, sequential fermentation *M. pulcherrima-S. cerevisiae*. Letters indicate significant differences in a row (one-way ANOVA and Tukey’s test, *p* < 0.05).

^1^Below detection limit (0.01 mg/L eq).

After 2 days of fermentation Mp and Td showed reduced levels of all the distinctive compounds that characterize the headspace. The levels of ethyl acetate were 20.45 and 15.16 mg/L eq., for Mp and Td, respectively, while other ethyl esters such as ethyl caproate, ethyl caprylate, ethyl caprate were only detected in trace amounts, with concentrations in the headspace ranging from 0.01 to 0.65 mg/L eq (Table 2). These samples revealed concentration of isoamyl alcohol at 10.91 (Mp) and 15.64 mg/L eq. (Td), respectively. Moreover, Mp was characterized by higher concentrations of isobutyl alcohol (4.29 mg/L eq.) and β-phenylethanol (3.04 mg/L eq.), whereas Td showed a higher concentration of β-Phenylethyl acetate (0.65 mg/L e), β-Phenylethanol (0.89 mg/Leq.), caproic and caprylic acids (1.04 and 4.11 mg/L eq, respectively), Amyl methyl ketone (2.09 mg/L eq.) and 1-propanol (4.28 mg/L eq.). Finally, the metabolic activity of Mp led to an accumulation of acetic acid of 2.16 mg/L eq., while this compound was less present in Td which exhibited a higher accumulation of caproic (1.04 mg/L eq.) and caprylic acids (4.11 mg/L eq.).

However, at the conditions adopted in this research, the effects of the sequential fermentations on VOCs were more evident after a prolonged incubation at 20°C. In fact, at the end of fermentation, all the samples exhibited 76.85–86.72 mg/L eq. of ethyl acetate, 1.71–3.95 mg/L eq. methyl isobutyl ketone, 62.70–78.81 mg/L eq. amyl methyl ketone, and 70.59–86.65 mg/L eq. isoamyl alcohol. The sequential fermentation achieved by inoculating Sc into batches fermented with Mp and Td allowed the diversification of volatile profiles in the samples after 10 days of fermentation. This observation is consistent with previous findings concerning the metabolic traits of the two non-*Saccharomyces* strains and with the manufacturer’s specification. The sequential fermentation with Mp determined a significant (*P* < 0.05) increase of esters, confirming the findings of other authors (Jolly et al., 2014; Zohre and Erten, 2002), with higher levels ethyl caproate, ethyl caprylate, ethyl caprate and β-phenylethyl acetate. These compounds are responsible for the fruity flavors of wines (tropical fruits and green apple) (Gamero et al., 2020) and their increase is usually due to higher medium-chain fatty acid production. These aromas can positively affect young wines aroma, especially in those with neutral flavors, thus suggesting a possible use of this yeast in preserving the quality of white and rosè wines by providing antioxidant activity.

*T. delbrueckii* provides wines with flower and sweet aroma, a reduced astringency and higher roundness degrading in part malic acid and releasing membrane polysaccharides. Therefore, it may be suggested in red wine production.

Indeed, Romano et al. (2022a), Ruiz et al. (2018) found that, when used in mixed grapevine must fermentation with *S. cerevisiae*, *M. pulcherrima* increases the production of medium-chain fatty acids (especially caprylic acid) and esters. Rollero et al. (2015) reported that the final concentrations of ethyl caproate and ethyl caprylate were positively correlated with the initial nitrogen content and negatively correlated with temperature and lipid content. Also, Berbegal et al. (2020) found that that the sequential fermentation of *M. pulcherrima* with *S. cerevisiae* resulted in a balanced VOC profile producing a richer and more complex aroma profile than either yeast alone. According to these Authors, the combination of *M. pulcherrima* with *S. cerevisiae* resulted in a balanced VOC profile, where the robustness of *S. cerevisiae* fermentation was complemented by the unique aromatic contributions of *M. pulcherrima*, highlighting ask key VOCs from this combination B-phenylethyl acetate and ethyl acetate.

Also, Td confirmed the good attitude in winemaking. The samples, in fact, were characterized, in comparison with Sc pure fermentation, by significantly higher levels of ethyl acetate (85.72 mg/L eq.), 1-propanol (17.36 mg/L eq.) and β-phenylethanol (14.79 mg/L eq.), by lower medium-chain fatty acid ethyl ester concentrations and the lowest level of acetic acid. Therefore, in these wines, aroma complexity was enhanced with floral notes due to the high concentrations of β-phenyl ethanol, which carries a pleasant odor reminiscent of roses, and a sweeter taste due to low acetic acid production. It is worth mentioning that β-phenyl ethanol could be also linked to variety contribution, and to some extent *M. pulcherrima* could increase its content due to its β-glucosidase activity; this aspect should be confirmed and validated in future research. Also, the data of the literature confirmed that, when used in sequential fermentation with *S. cerevisiae*, *T. delbrueckii* increased the amounts of volatile esters (especially ethyl acetate) (Ivit et al., 2020) as well as the amounts of higher alcohols including 1-propanol and β-phenylethanol (Azzolini et al., 2015; Comitini et al., 2011) and reduced the overall acetic acid content in wine (Arslan et al., 2018; Azzolini et al., 2015; Belda et al., 2015, 2017; Comitini et al., 2011; Ramírez and Velázquez, 2018) with an a positive impact on the sensory properties of wine. The combination of aromatic notes, as well as the reduced astringency, a higher roundness (malic acid degradation and polysaccharides release) suggests the possible use of this kind of sequential fermentation for red wines.

### 3.6 Amino acids and VOCs

The last research question was on a possible correlation between amino acids and VOCs, as several authors reported a strong correlation between amino acids and aroma compounds (Fairbairn et al., 2017). In particular, the effect of single amino acids on major volatile production by two *S. cerevisiae* strains was evaluated in synthetic grape must and it was found a linear correlation between the amino acid concentration and the corresponding volatile compound, resulting in the predictable production of aromatic compounds (Fairbairn et al., 2017). A possible approach is to perform a correlation between each amino acid and its deriving aroma compounds; however, this approach could be fail-dangerous (that is the prediction results in lower values than the effective ones), because it does not consider the possible correlations amongst different amino acids (Fairbairn et al., 2017), and the fact that an amino acid could be a precursor for several aroma compounds.

**FIGURE 7:**
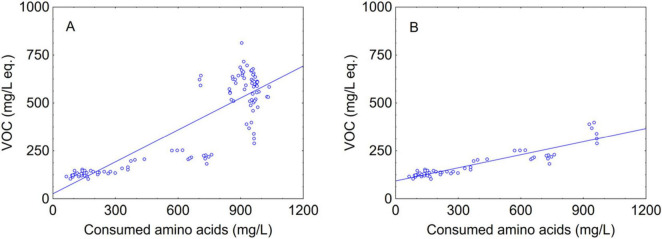
Scatter plot of consumed amino acids vs VOC. **(A)** total; **(B)** after 2 days of fermentation. The line represents the linear fitting.

Therefore, in this paper a novel approach was proposed, that is the correlation of total amino acid amount vs total VOCs concentration (Figure 7A); this method suggests at least a partial correlation (R^2^ at 0.750, *P* < 0.01). Moreover, when the same approach was applied separately to the data after 2 days of fermentation there was a higher correlation with an R^2^ coefficient of 0.839 (*P* < 0.01) (Figure 7B), and to the best of authors’ knowledge this is a novelty compared to existing literature because it could contribute to give an overview on the impact of amino acid metabolism not at the end but during the fermentation. Indeed, at this stage of fermentation the aromatic amino acids (phenylalanine, tryptophan, tyrosine), the branched-chain amino acids (isoleucine, leucine, valine) and methionine and threonine, which are precursors to higher alcohols via Erlich pathway, were consumed by Mp and Td at high percentages, thus confirming the importance of focusing on amino acid metabolism of non-*Saccharomyces* yeasts immediately before *S. cerevisiae* inoculation to clearly demonstrate their contribution to the aroma. Higher alcohols may be involved in the formation of esters, thus, influencing the profile of volatile compounds in wine. Although VOCs composition and concentrations are the result of other metabolic pathways and a multitude of factors (Dzialo et al., 2017), a strong relationship between amino acid content in must and fermentation volatile compounds has been demonstrated by different studies (Beltran et al., 2005; Hernández-Orte et al., 2005; Lu et al., 2023).

**FIGURE 8:**
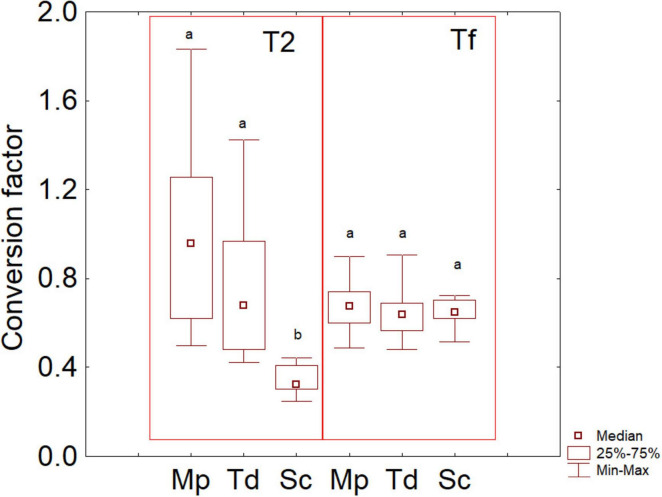
Box-whisker plot on the conversion factor consumed amino acids vs VOC. The letters indicate significant differences (Friedman test, *P* < 0.05). Sc, pure fermentation of *S. cerevisiae*; Td, sequential fermentation *S. cerevisiae-T. delbrueckii*; Mp, sequential fermentation *S. cerevisiae-M. pulcherrima*. T2, after 2 days; Tf, final time of fermentation.

To corroborate the idea of a possible correlation between amino acids and aroma compounds, a conversion factor was evaluated as VOCs vs consumed amino acids; then, these indices were used to build a box-whisker plot (Figure 8). This approach is complementary to the correlation analysis, as an index of 1 means a 100% correlation.

After 2 days, Mp and Td had median conversion coefficients of 0.958 (first and third quartiles at 0.619 and 1.256, that is a correlation of at least 61%) and 0.681 (first and third quartiles at 0.478 and 0.979) respectively, while a significantly lower factor was found for Sc (median at 0.324, and quartiles at 0.302 and 0.410). On the other hand, at the end of the trials, the median conversion factors did not show significant differences, being 0.676 for Mp, 0.638 for Td and 0.650 for Sc.

## 4 Conclusion

This investigation described a comprehensive approach focusing on enological performances of two non-*Saccharomyces* starter cultures, that is Mp (*M. pulcherrima*, LEVULIA^®^PULCHERRIMA), Td (*T. delbrueckii*, LEVULIA^®^ TORULA), used in sequential fermentations with Sc (*S. cerevisiae* FERMOL^®^ Red Fruit). The findings suggest that using non-*Saccharomyces* yeasts in sequential fermentations may be a valuable method for producing wines with distinct characteristics and with greater aromatic intensity and complexity than those obtained from *S. cerevisiae* single-culture fermentation.

Furthermore, understanding which amino acids are taken up during the initial stages of alcoholic fermentation could be beneficial for optimizing nitrogen sources in successive fermentations. In addition, from a practical point of view, the paper offers a protocol, in terms of high number of independent fermentations (six units performing experiments), and a possible approach for data treatment (standardization per units of cells for some metabolisms, correlation between possible substrates and products, focus on the whole dataset rather than on single parameters) to propose an effective scalability and application of *Saccharomyces* and non-*Saccharomyces* yeasts. The use of commercial strains, with well-known characteristics, and of a commercial grape-must, was a convenient strategy to avoid the confounding effect due to matrix and microorganisms.

Further experiments are necessary to scale up and validate the observations and results hereby collected at industrial level; first, sensory analyses are required to effectively correlate the findings in terms of aroma and medium composition with the sensory notes of produced wines, as this is the first trait required by wine producers. In addition, the effect of large volumes, and enological practices should be assessed too. However, this research offers a contribution to the topic of sequential fermentation non-*Saccharomyces*/*Saccharomyces* yeasts and stresses the importance of holistic approaches when designing new fermentation strategies.

## Data Availability

The raw data supporting the conclusions of this article will be made available by the authors, without undue reservation.
