# Supplementary material for: Comparison between Metschnikowia pulcherrima and Torulaspora delbrueckii used in sequential wine fermentations with Saccharomyces cerevisiae
Source: Front Microbiol. 2025 Jul 2;16:1590561. doi: 10.3389/fmicb.2025.1590561 (PMC12263952; doi:10.3389/fmicb.2025.1590561)
Supplement: Supplementary file 1 [file Table_1.docx]

**Comparison between *Metschnikowia pulcherrima* and *Torulaspora delbrueckii* used in sequential wine fermentations with *Saccharomyces cerevisiae***

Lisa Granchi*, Francesca Patrignani*, Angela Bianco, Giacomo Braschi, Marilena Budroni, Laura Canonico, Angela Capece, Anna Cauzzi, Maurizio Ciani, Fabio Chinnici, Valentina Civa, Luca Cocolin, Paola Domizio, Vasileios Englezos, Nicola Francesca, Carmela Gerardi, Francesco Grieco, Rosalba Lanciotti, Silvia Mangani, Carlo Montanini, Vincenzo Naselli, Giorgia Perpetuini, Rocchina Pietrafesa, Angela Racioppo, Gabriella Siesto, Rosanna Tofalo^2^, Antonio Bevilacqua**, Patrizia Romano**

***Equal contributions:** these authors equally contributed to the work

****Corresponding authors:** A. Bevilacqua ([antonio.bevilacqua@unifg.it](mailto:antonio.bevilacqua@unifg.it)), P. Romano ([patrizia.romano@unimercatorum.it](mailto:patrizia.romano@unimercatorum.it))

**SUPPLEMENTARY MATERIAL**

**Table S1:** Technical sheet of yeasts

| **Commercial name** | **Species and characteristics** |
| --- | --- |
| **FERMOL® Red Fruit** | *Saccharomyces cerevisiae*  Yeast strain obtained from the hybridization of two strains: Fermol Iper R x Fermol 2033.  High fermentative vigour, which is also kept under stress conditions such as in musts with a high sugar content.  It can be used for red wines, where aromatic notes such as blueberry, black currant and raspberry should be highlighted.  https://www.aeb-group.com/en/fermol-red-fruit-4742 |
| **LEVULIA® TORULA** | *Torulaspora delbrueckii*  Ability to limit the development of volatile acidity-producing species.  It is suitable for both terpene and thiol grape varieties (Sauvignon Blanc, Chardonnay, Gewurztraminer, Colombard, Riesling, Muscat, Sémillon, etc.). It greatly enhances aromatic expression in wines by improving balance and complexity. In addition, the release of nutrients and polysaccharides contributes to a reduction in astringent notes.  https://www.aeb-group.com/media/catalogo-unico/levulia_torula-2288/docs/en/LEVULIA_TORULA_TDS_EN_1090421_OENOLIA_Italy.pdf |
|  | *Metschnikowia pulcherrima*  Ability to produce compounds of oenological interest such as esters, thyols and produces minimal undesirable compounds such as acetic acid  https://www.aeb-group.com/media/catalogo-unico/levulia_pulcherrima-2888/docs/en/LEVULIA_PULCHERRIMA_TDS_EN_1190421_OENOLIA_Italy.pdf |

**Table S2A:** Amino acid concentration (mg/L) (mean values±standard deviation) of the commercial grape must before fermentation (T0) and after 48 h of fermentation with *M. pulcherrima* (Mp), *T. delbrueckii* (Td) or *S. cerevisiae* (Sc). Letters indicate significant differences (one-way ANOVA and Tukey’s test, P<0.05).

| **Amino acid** | **T0** | **Mp** | **Td** | **Sc** |
| --- | --- | --- | --- | --- |
| Alanine | 44.47 ±4.48a | 35.38 ± 6.00a | 16.93 ± 9.53 b | 8.33 ± 3.90 c |
| Arginine | 678.88 ± 54.39a | 644.68 ± 32.76a | 598.44 ± 62.12a | 197.45 ± 127.44b |
| Asparagine | 17.29 ± 2.88a | 12.75 ± 1.55 b | 3.14 ± 1.46 c | 1.90 ± 0.84 c |
| Aspartate | 19.47 ± 2.13a | 16.55 ± 4.35 a | 2.34 ± 1.58 b | 2.84 ± 0.81 b |
| Glutamine | 14.36 ± 2.14a | 8.15 ± 3.11b | 5.35 ± 2.82c | 5.82 ± 2.27b |
| Glutamate | 28.74 ± 4.84a | 18.44 ± 2.67b | 9.09 ± 5.31c | 4.61 ± 1.30c |
| Glycine | 5.28 ± 1.10a | 4.46 ± 0.98a | 4.91 ± 1.26a | 3.22 ± 1.87a |
| Histidine | 13.20 ± 3.46a | 13.19 ± 0.85 a | 10.21 ± 1.77a | 3.04 ± 1.39b |
| Leucine | 21.95 ± 2.91a | 14.71 ± 3.53b | 2.64 ± 1.82c | 1.29 ± 0.96c |
| Lysine | 14.24 ± 3.40a | 8.55 ± 2.16b | 2.72 ± 4.08c | 5.42 ± 2.92c |
| Methionine | 2.48 ± 0.65a | 0.76 ± 0.25b | 2.47 ± 0.85c | 0.63 ± 0.55b |
| Phenylalanine | 17.46 ± 2.91a | 14.14 ± 1.98a | 4.81 ± 2.82b | 0.82 ± 0.64c |
| Serine | 25.41 ± 4.99a | 24.45 ± 3.60a | 4.76 ± 1.66b | 1.61 ± 0.70c |
| Threonine | 19.68 ± 3.20a | 12.04 ± 1.88b | 2.69 ± 2.36c | 2.90 ± 1.22c |
| Tyrosine | 16.16 ± 3.47a | 14.65 ± 1.44a | 6.20 ± 3.54b | 1.19 ± 0.51c |
| Valine | 18.86 ± 2.51a | 16.81 ± 2.28a | 7.18 ± 3.40b | 5.36 ± 1.75b |
| Isol+Tryptophan | 14.72 ± 1.62a | 10.74 ± 2.44a | 1.94 ± 1.27b | 0.93 ± 0.49b |
| Proline | 225.50 ± 10.28a | 225.44 ± 13.37a | 216.33 ± 14.55a | 222.03 ± 14.49a |

**Table S2B:** Amino acid composition (mg/L) (mean values±standard deviation) at the end of both sequential fermentations carried out by *M. pulcherrima/S. cerevisiae* (Mp) and *T. delbrueckii/S. cerevisiae* (Td) and in pure culture by *S. cerevisiae* (Sc).

| **Amino acid** | **Mp** | **Td** | **Sc** |
| --- | --- | --- | --- |
| Alanine | 12.87±4.96 | 7.47±5.06 | 4.50±5.42 |
| Arginine | 35.82±23.72 | 14.24±9.97 | 7.77±4.25 |
| Asparagine | 3.56±0.92 | 1.97±0.90 | 2.60±0.80 |
| Aspartate | 1.39±0.76 | 0.91±0.30 | 1.88±0.96 |
| Glutamine | 3.18±0.58 | 2.91±1.57 | 2.94±2.28 |
| Glutamate | 4.41±1.49 | 3.04±1.81 | 2.96±2.42 |
| Glycine | 0.61±0.28 | 0.37±0.31 | 0.89±0.53 |
| Histidine | 3.28±1.84 | 1.52±1.46 | 0.89±0.42 |
| Leucine | 2.01±1.37 | 0.66±0.39 | 1.86±1.61 |
| Lysine | 2.54±0.77 | 2.09±1.86 | 3.05±2.18 |
| Methionine | 0.34±0.13 | <0.1 | 0.57±0.24 |
| Phenylalanine | 1.14±0.95 | 0.61±0.50 | 1.12±0.95 |
| Serine | 1.15±0.49 | 1.19±0.54 | 1.32±0.56 |
| Threonine | 4.32±1.61 | 0.65±0.36 | 1.34±0.86 |
| Tyrosine | 0.99±0.42 | 0.93±0.46 | 0.98±0.50 |
| Valine | 0.95±0.56 | 0.33±0.37 | 0.90±0.68 |
| Isol+Tryptophan | 0.58±0.31 | 0.37±0.30 | 0.57±0.30 |
| Proline | 232.29±25.33 | 220.06±17.94 | 223.35±15.32 |

**Table S3:** Amounts of phenolic compounds (mg OE/L) (mean values±standard deviation) at the end of both sequential fermentations carried out by *M. pulcherrima/S. cerevisiae* (Mp) and *T. delbrueckii/S. cerevisiae* (Td) and in pure culture by *S. cerevisiae* (Sc). ND, not detected. T0, commercial grape must before fermentation.

Letters indicate significant differences (one-way ANOVA and Tukey’s test, P<0.05).

|  | T0 | Mp | Td | Sc |
| --- | --- | --- | --- | --- |
| Quercetin | 0.38±0.04a | 0.37±0.03a | 0.34±0.03a | 0.35±0.05a |
| Gallic acid | 6.27±0.16a | 10.58±0.61c | 9.01±0.49b | 10.20±0.53c |
| Catechin | 5.69±0.48a | 5.62±0.42a | 5.27±0.52a | 5.51±0.63a |
| Malvidin-3-O-glucoside | 13.99±2.29a | 10.72±1.57b | 10.37±1.91b | 10.71±1.99b |
| Trans-resveratrol | ND | ND | ND | ND |
| Trans-coutaric acid | 6.84±0.35a | 6.32±0.20b | 5.99±0.28c | 6.17±0.24b,c |
| Caftaric acid | 42.42±1.56a | 38.37±1.36b | 36.62±1.47c | 37.501.42b,c |
| Caffeic acid | 1.14±0.07a | 1.42±0.06b | 1.36±0.08bb | 1.44±0.07 |
| Kanferolo-3-O-glucoside | 0.56±0.08a | 0.46±0.12a,b | 0.42±0.07b | 0.44±0.10a,b |
| Cyanidin-3-O-glucoside | 0.35±0.05 | ND | 0.12±0.01 | ND |
| Quercetin-3-O-glucoside | 4.46±0.34a | 3.48±0.25b | 3.48±0.34b | 3.34±0.43b |
| Rutin | 0.82±0.08a | 0.99±0.21a | 1.00±0.20a | 0.91±0.20a |
| Total anthocyanins | 34.40±5.00a | 20.99±2.94b | 22.04±4.19b | 22.55±6.41b |
